# Recent Advances in Cancer Plasticity: Cellular Mechanisms, Surveillance Strategies, and Therapeutic Optimization

**DOI:** 10.3389/fonc.2020.00569

**Published:** 2020-04-22

**Authors:** Giuseppe Nicolò Fanelli, Antonio Giuseppe Naccarato, Cristian Scatena

**Affiliations:** Division of Pathology, Department of Translational Research and New Technologies in Medicine and Surgery, University of Pisa, Pisa, Italy

**Keywords:** cancer plasticity, stem cell, heterogeneity, recurrence, liquid biopsy

## Abstract

The processes of recurrence and metastasis, through which cancer relapses locally or spreads to distant sites in the body, accounts for more than 90% of cancer-related deaths. At present there are very few treatment options for patients at this stage of their disease. The main obstacle to successfully treat advanced cancer is the cells' ability to change in ways that make them resistant to treatment. Understanding the cellular mechanisms that mediate this cancer cell plasticity may lead to improved patient survival. Epigenetic reprogramming, together with tumor microenvironment, drives such dynamic mechanisms favoring tumor heterogeneity, and cancer cell plasticity. In addition, the development of new approaches that can report on cancer plasticity in their native environment have profound implications for studying cancer biology and monitoring tumor progression. We herein provide an overview of recent advancements in understanding the mechanisms regulating cell plasticity and current strategies for their monitoring and therapy management.

## Cancer Cell Plasticity: a New Level of Heterogeneity in a Tumor

Tumor heterogeneity can be inter-tumoral, if genetic variations are found among different patients with tumors of the same type, or intra-tumoral, involving different cancer cells in the same tumor. In particular, intra-tumor heterogeneity can be caused by genetic variation, modulation in the expression of a gene, transition among cellular states or environmental changes (1). Thus, it is easy to understand that intra-tumor heterogeneity drives cancer progression and represents the main cause of treatment failure (2).

**Figure 1:**
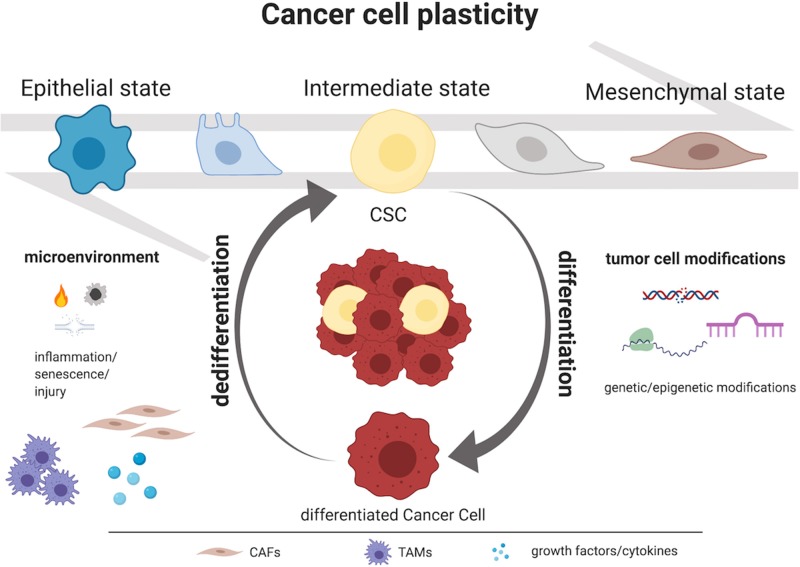
Mechanisms governing CSC plasticity model. Intra-tumor heterogeneity relies on the capacity to shift dynamically and reversibly between CSC and non-CSC/differentiated state. Tumor cell modifications as genetic and epigenetic alterations and microenvironment perturbations as inflammation, injury, and senescence represent the major causes of cancer cells plasticity. Moreover, CSCs exhibit an induced epithelial-to-mesenchymal transition (EMT) program and, particularly, they display an intermediate state of EMT. This process depends on both genetic mutations, epigenetic modifications and transcriptional modulation of cancer cells and signals provided by the tumor microenvironment (i.e., growth factors, cytokines, CAFs or TAMs). Created with BioRender.com.

Initially, two models were proposed to justify intra-tumor heterogeneity: the “clonal evolution” model and the “cancer stem-like cell” (CSC) model. The first contemplates differences among cancer cells due to stochastic alterations in genes; according to this theory, clones which gain a growth advantage are selected over time (3, 4). The second involves CSCs, a minority population of cancer cells with self-renewing capacity that initiates and maintains tumor growth, in contrast with the majority of the cancer cells which show a more differentiated phenotype (5–7). Lately, a third model has been proposed: the “CSC plasticity” model, where CSCs possess the capacity to move between stem and differentiated states. This shift may be caused by intrinsic cues such as genetic mutations and/or epigenetic modifications but also by extrinsic cues from the tumor microenvironment (inflammation, injury, senescence). In addition, the tumor-initiating potential is enhanced by the overexpression of transcription factors involved in the process of epithelial-to-mesenchymal transition (EMT) (8–10) and CSCs exhibit an induced EMT program (11). These data suggest that EMT is strictly linked to CSC features. Indeed, CSCs switch between epithelial and mesenchymal states and this process depends on both genetic mutations, epigenetic modifications and transcriptional modulation of cancer cells and signals provided by the tumor microenvironment through the mediation of growth factors, cytokines, cancer-associated fibroblasts (CAFs), tumor associated macrophages (TAMs) and hypoxia (12, 13) (Figure 1). These transitions promote metastasis at distant sites as well as drug resistance and, therefore, disease recurrence (14–16). In breast cancer cells co-expression of epithelial and mesenchymal genes promotes stemness inducing the formation of 3D-spheroid structures named “tumor-spheres” (17). Moreover, cells with intermediate state of EMT showed similar tumor-initiating potential when compared with fully differentiated mesenchymal cells in a mouse model of prostate cancer (18). Thus, we suppose that cancer cell stemness may be associated with a partial EMT phenotype and, indeed, cells which exhibit this intermediate EMT state possess a much more pronounced plasticity (19). According to this definition, the CSC plasticity model suggests that the two historical models of cancer heterogeneity, i.e., the clonal evolution model and the CSC model, are not mutually exclusive (1, 20–22). We believe that this third model suggests a new level of complexity in tumor heterogeneity concept.

## Novel Approaches for Monitoring Tumor Cell Plasticity and Progression

Solid tumors are consisted of several sub-clonal cells populations, which compete in a Darwinian manner under the selective pressures of endogenous and exogenous factors, leading to the clonal evolution of a dominant subclone that will characterize the tumor's molecular landscape. Hence, it will be highly heterogeneous and will dynamically change during the disease progression, so longitudinal sampling is essential to define therapeutic strategies.

Currently, cancer molecular profile is evaluated through “solid biopsies” from primary tumor or metastatic nodule; however, this approach has several issues: (i) biopsies are not representative of the whole tumor mass; (ii) often tumor site is not accessible; (iii) frequently, biopsies cannot be serially performed; (iv) each metastasis could have a different genomic landscape; (v) finally, therapeutic selective pressure has to be considered too (23–28). To overcome these limitations the novel approach of “liquid biopsy” is gaining attention.

The rapid turnover of cancer cells results in the constant release into the bloodstream of: (i) cell-free circulating tumor DNA (ctDNA); (ii) tumor derived RNA (predominantly micro-RNAs and long-non-coding-RNA) (29); (iii) circulating tumor cells (CTCs); and (iv) extracellular vesicles (EVs) (sub-cellular structures with a membrane that contain nucleic acids and/or proteins) (30–33). This enables clinicians to repeatedly and non-invasively interrogate the dynamic evolution of human cancers.

CTCs are (probably) intravasated or passively spread from the primary and/or secondary tumor sites into the bloodstream, and could be responsible for the beginning of distant metastases.

In cancer patients CTCs can be isolated single or in clusters with other CTCs or with endothelial cells, platelets, leukocytes and fibroblasts, conferring them resistance to oxidative stress, and protection from the immune system (34). Their absolute number is really low (~1 CTC per 1 × 10^9^ blood cells), especially in early cancer stage, and can vary between cancer types (34–36).

CTCs detection and isolation challenges are related to the high sensitivity and specificity required, and several factors still hamper standardized clinical application. Different approaches have been extensively investigated to isolate CTCs: (i) technologies such as density gradient stratification, membrane filtration, photoacoustic detection, dielectric mobility, and microfluidic separation are based on CTCs physical properties (density, size, mechanical plasticity, and dielectric mobility) that are different from those of other blood cells (37, 38). However, these techniques have low specificity (39), so new antibody-based functional assays have been developed: (ii) cytometric high-throughput imaging which provides the scanning of cells on slides; (iii) negative depletion of leucocytes and erythrocytes (Batch cell lysis, Microfluidic CTC-iChip, Immunomagnetic separation) (40) using specific antigens such as CD45 for leucocytes and glycophorin for erythrocytes; (iv) positive CTC enrichment by specific markers expressed on the cell surface (CellSearch, Magsweeper, Microfluidic CTC-Chip) such as epithelial cell adhesion molecule (EpCAM) (41) cytokeratins (CK8, CK18, CK19) (42) or tumor specific markers (TTF-1, PSA, HER-2 etc.) (43, 44). Nevertheless, no agreement has been reached on the specific antibodies to test. Indeed, EpCAM is usually lost during EMT, that sustains CTC migration, extravasation and apoptosis/anoikis resistance (45). Additionally, CTCs may develop a stem-like phenotype (46, 47). Hence, it is possible to find “commingling” CTCs that express epithelial, EMT or cancer stem cells phenotype; these CTCs have the highest plasticity potential and thus may represent CSCs (48). Different expression levels of stem cell markers such as CD24, CD44, CD133, ALDH, NANOG, OCT4, were found in ovarian (49), breast (50), and prostate CTCs (51). Remarkably, CTCs differentially express genes involved in oncogenic signaling pathways depending on their plasticity or stemness levels (52–54).

Finally, innovative developed approaches to CTCs/CSCs isolation are based on: (v) CTCs functional features such as protein secretion and cell migration (Epispot assay, Invasion assay) that allow the attachment of these cells to synthetic substrates co-treated with specific matching molecules (55); (vi) nanotechnology (Immunomagnetic nanobeads, Nanostructures substrates in microchip) (56, 57); (vii) the combination of surface/cytoplasmic markers, size and dielectrophoretic migration properties (DEPArray) (58).

Despite different approaches, in our opinion none of them completely satisfy the necessary requirements since low purity, loss of CTCs, and a narrow detection range still need to be tackled.

Finally, an additional central aspect to consider in the cancer plasticity is the complex network of epithelial-stromal cells interactions. Stroma undergoes, in parallel with the epithelial compartment, in a dynamic remodeling that may predict and explain several clinico-pathological features (59–63). To date, several *in-vitro* and in *in-vivo* models have been created and novel approaches have been used to study this interaction and its remodeling (64–66): genomic (scRNA-seq); protein translation and secretion (serial analysis of gene expression, antibody arrays and bead-based arrays, mass spectrometry and yeast, bacterial and mammalian secretion traps); autocrine, paracrine and long distance (cells co-culture, proximal culture); and directly in human tissue (multispectral imaging analysis). However, stroma characterization is still incomplete and fragmentary, also because of the difficulty to perform an “evolution tracking” of the whole stromal compartment.

Since malignancies development and progression are the result of these complex interactions, we believe that the treatment with chemotherapeutic agents against the cancer epithelial compartment combined with novel stroma-targeted therapies, may efficiently reduce cancer recurrence, also thank to the targeting and eradication of CSCs.

## Clinical Relevance of Cancer Cell Plasticity: Limitations and New Opportunities

Though the presence of CTCs has been known since the 1869 (67), their clinical relevance was demonstrated only in 1994 (68). Despite their low number in the blood stream, they are related to clinical outcomes (34–36). In our opinion CTCs and CSCs may represent the key for early diagnosis, better prognostic stratification and a more accurate therapeutic response prediction; in addition, their concentration and pheno/genotyping could be easily measured and repeated over time. To date, however, only few authors tried to demonstrate advantages of liquid biopsy over the solid biopsies in cancer surveillance and follow-up (69, 70); this is also due to the important technical issues still to be overcome. In addition, according to recent insights, CSCs do not constitute an autonomous compartment; rather, they play an active role in the microsystem, constituted both by the epithelial and the stromal compartments; indeed several authors have demonstrated the mutual influences between CSCs and their microenvironment (71–74).

We think that one promising approach to eradicate CSCs may be to target the EMT (75): inhibitors of TGFβ-induced EMT as well as SRC, MEK, or ALK5 inhibitors have been tested (76, 77). Interestingly, also inflammatory cytokines—IL6 and IL8 in particular—may represent potential therapeutic targets of EMT: IL-6 acts as a direct regulator of breast CSCs (BCSCs) self-renewal (78) and high levels of IL-6 are demonstrated to be associated to poor clinical outcome (79); on the other hand, BCSCs have been successfully eradicated both *in vitro* and in animal models by blocking the IL-8 receptor CXCR1 (80). In addition, in patients with HER2 positive breast cancer, treatment with HER2 inhibitors decreased the content of BCSCs (81), suggesting that combination therapies that include HER2 targeting agents may overcome BCSCs resistance. Based on this knowledge, we believe that therapies targeting BCSCs represent an urgent need to prevent recurrence. Other authors have suggested to target also Notch, Hedgehog, Wnt and PI3K/Akt/mTOR pathways (82). Intriguingly recent evidences demonstrate that CSCs rely on mitochondrial biogenesis for their propagation (83). Lamb et al. previously demonstrated that the antibiotic doxycycline, in a known inhibitor of the 28S mitochondrial ribosome subunit, inhibits CSC propagation *in vitro* (84). In 2018 we performed a pilot clinical trial and demonstrated that doxycycline treatment decreases the expression of CSC markers in breast cancer tumor samples (85). We thus propose that selected antibiotics, in monotherapy or in combination, may be further studied as interesting drugs for the eradication of CSCs.

From now on, this review concentrates on specific issues concerning cancer cell plasticity in breast cancer, glioblastoma, and melanoma, which represent our expertise and, in our opinion, the most challenging models in this field. A detailed table is then provided reporting the latest knowledge in other tumor models.

## CSC Plasticity in Breast Cancer

Breast cancer has been largely investigated in terms of its etiology (86–89) and still little is known on the mechanisms of its progression. Breast cancer cells commonly gain genetic and epigenetic modifications in their genome (90), contributing to its characteristic intra-tumor heterogeneity (91–96). Intra-tumor heterogeneity is strongly influenced by numerous factors from the tumor microenvironment: breast cancer cells are indeed under continuous selective pressure due to attacks by the immune system or administered therapies (97, 98). This supports breast cancer progression, conferring a competitive advantage to specific subclones (92).

In recent decades, a hierarchical organization has been proposed, where cancer cells with self-renew capacity, the so-called BCSCs, are postulated to be at the top of the tumor pyramid. Al-Hajj et al. in 2003 first isolated a population of BCSCs expressing high levels of CD44 and low levels of CD24 (CD44^+^CD24^−/low^) and capable to form tumors when injected into immune deficient mice (99). Since then, numerous studies have tested other biomarkers to sort BCSCs: among all, aldehyde dehydrogenase 1 (ALDH1) resulted to be a potentially useful alternative or complement to the CD44^+^CD24^−/low^ phenotype, particularly in high grade and HER2 positive tumors (100). BCSCs not only possess high tumorigenic properties but represent the cells that mediate tumor metastasis. Indeed, the CD44^+^/CD24^−/low^ phenotype is highly expressed in triple negative breast cancers (101, 102) and is associated to poor overall survival (103, 104); moreover, it has been reported among cancer cells spread into the bone marrow (105) or to the lung (106) of patients with breast cancer. At present, BCSCs are believed to enter the circulation and become CTCs: indeed, high expression levels of BCSC markers have been found in CTCs (107). Thanks to their capacity of anoikis resistance, CTCs with BCSC phenotype have the potential to seed metastatic lesions (108). Studies from liquid biopsy samples demonstrate that CTCs with a BCSC phenotype are enriched in the group with clinical disease progression (107).

A large number of studies also suggest that BCSCs display resistance to traditional cancer therapies (109–116). Cytotoxic chemotherapies target the bulk of the tumor composed of highly proliferative breast cancer cells and does not affect BCSCs that, over time, cause tumor relapse (81). In addition, genetic alterations may confer to BCSCs intrinsic chemoresistance, including modifications in proteins involved in the detoxification of chemotherapy agents (117). As reported above, BCSCs express high levels of ALDH1, that metabolizes cyclophosphamide, thus minimize its toxic effects (101). Also, tumor microenvironment plays a crucial role in BCSC chemoresistance: in hypoxic conditions, activation of hypoxia induced factors not only promotes the formation of new blood vessel but also a BCSCs quiescent phenotype (118, 119).

## CSC Plasticity in Glioblastoma

Glioblastoma (GBM) is the most frequent and deadly glial tumor (120); it is morphologically (121) and molecularly (97, 122, 123) characterized by high intra- and inter- tumor heterogeneity, which may play a pivotal role in recurrence and therapy resistance (124, 125).

The Cancer Genome Atlas has identified four GBM molecular subtypes: proneural, neural, classical, and mesenchymal (126). However, it has been demonstrated how multiple molecular subtypes may co-exist in the same tumor mass (122) or how GBM presents hybrid states with the expression of a peculiar signature overlapping two molecular subtypes (127). The establishment and the constant evolution of this heterogeneity equilibrium are due to glioma stem cells (GSCs) (128) and can be influenced by cytotoxic therapies and other endogenous factors (129, 130). However, how GSC heterogeneity is determined still remains unclear; *in-vitro* studies have shown that GSCs preserve their capability for recapitulating their primary heterogeneity also after many cell divisions, and temozolomide (TMZ) does not influence this capacity (131, 132); though, the same cytotoxic drug is able to drive GSCs heterogeneity and further drug resistance (133).

GSCs' isolation and characterization are based on stem markers expression; therefore, their choice is fundamental. One of the first discovered marker was CD133 (134); however, its expression is highly variable (~20–60%) (135), and also CD133– cells have a clonogenic potential. Indeed, Chen et al. (136) divided GSCs into three subtypes based on malignant potential (MP): type 1 (high MP) and type 3 (mild MP) were CD133−; whereas, type 2 GSCs (moderate MP) were CD133+. An additional marker is CD15, which is more frequently expressed in GBM than CD133; CD15+ GSCs are more clonogenic, proliferative and tumorigenic (137). CD44 represents another reliable marker: indeed, CD44+ GSCs present high tumor-sphere forming and tumorigenic potential, and have the capability to restore the heterogeneity of the parental GBM (138). Furthermore, ALDH1A3+ GSCs, besides having the above mentioned features, express other stem cell markers, such as musashi and nestin, and are able to differentiate into several neural lineages (139, 140), and promote TMZ resistance (141).

Nevertheless, a clear-cut segregation of GBM cells between CSCs and non-CSCs is not possible yet; instead, it is more conceivable the ability of GBM cells to transit among states or the acquisition of intermediate or metastable cellular state, exhibiting a wide and continuous range of CSC signature (142, 143).

## CSC Plasticity in Melanoma

Melanoma represents a significant challenge, with low curative rates (<10%) and poor prognosis (median survival: 6–9 months) in the metastatic stage (144–146). Aggressive melanoma has revealed to co-express specific genes and proteins of multiple cellular types, including embryonic stem cells and endothelial cells, underlying cell plasticity.

3D *in vitro* models demonstrated that melanoma cells are able to form perfusable, vasculogenic-like channels, a biological phenomenon called vasculogenic mimicry (VM) (147). The treatment with endostatin has proved no effect on the inhibition of melanoma VM (148), thus portraying aggressive melanoma as being able to survive by its own perfusion network (149).

On the other hand, a large number of molecular studies jointly revealed a strong stem signature in aggressive melanoma, with still unknown practical significance (150–152). In particular, Nodal, a signaling pathway active in embryonic development, was notably upregulated in more aggressive melanoma (153). The nodal family of proteins, are a subset of the TGFβ superfamily and cooperate to the pluripotency of human embryonic stem cells (154). This observation led researchers to recognize a commonality in the phenotype of aggressive melanoma, linking vascular, embryonic and cancer stem cell properties.

## CSC Plasticity in Other Solid Tumors

Several authors have demonstrated how it is possible to isolate CSCs in most solid malignancies. However, several aspects and molecular features regarding cell stemness still remain uncovered; this means that even if most markers across different cancer are the same (Table 1), a common and reliable signature is still lacking, due to technical issues mostly. Nevertheless, in our opinion, a change in clinical trials approach may be of help to overcome this limitation. Indeed, the implementation of biobanks of fresh tissues and biological fluids may represent a precious source for the next future when new techniques and novel approaches will be introduced.

**Table 1:** CSCs markers in other solid tumors.

**Type of cancer**	**CSCs Markers**	**References**
Head and neck squamous cell carcinoma	ALDH1, BMI1, c-MET, CD44, CD133	(155–159)
Lung cancer	ALDH1A1, ABCG2, BMI1, CD44, CD133, CD87, CD90, CD166, EpCAM, NANOG, NUCLEOSTEMIN, OCT4, PODXL-1, SOX2	(117, 160–163)
Esophageal carcinoma	ALDH1, ABCG2, CD13, CD44, CD90, CD271, INTEGRIN7, ICAM1, LGR5, SOX9	(164)
Gastric cancer	BMI1, CD44, CD54, CD71, CD90, CD133, CD166, LGR5, MUSASHI-1, OCT4, SOX2	(165)
Hepatocellular carcinoma	CD13, CD24, CD34, CD90, CD133, EpCAM, OV-6, SOX9, SOX12	(166–169)
Pancreatic cancer	ALDH1, c-MET, CD24, CD44, CD133, CXCR4, DCLK1, EpCAM, Lgr5	(170)
Colon cancer	ALDH1, CD26, CD29, CD44S, CD166, CXCR4	(171, 172)
Prostate cancer	ALDH7A1, ATXN1, CD24, CD44, PTEN, CD133, GATA3, KLF4, MYC, NKX3-1, TACSTD2, TNFSF11, TNFRSF11B	(50, 52, 173)
Ovarian cancer	ALDH1A1, c-MYC, CD24, CD44, CD117, CD133, CD243, CD338, EpCAM, IL-17R, LIN28, NANOG, OCT4, ROR1, SOX2	(49, 174–180)

*Most stemness markers are the same but a universal signature is still lacking*.

## Conclusions and Future Directions

Future research studies will be needed in order to improve our understanding of the complex phenomenon of cancer cell plasticity. The recent insights on the role of plasticity in cancer progression and relapse highlights the need to develop new and combinatorial therapies, that aim to: (i) inhibit specific cell markers; (ii) interfere with stemness and EMT signaling pathways; (iii) affect also components of the tumor microenvironment.

## Author Contributions

GF and AN wrote the paper. CS conceived the idea, supervised, and edited the manuscript. All authors discussed and commented on the manuscript.

## Conflict of Interest

The authors declare that the research was conducted in the absence of any commercial or financial relationships that could be construed as a potential conflict of interest.
